# Dimethyl Fumarate and Monoethyl Fumarate Exhibit Differential Effects on KEAP1, NRF2 Activation, and Glutathione Depletion *In Vitro*

**DOI:** 10.1371/journal.pone.0120254

**Published:** 2015-03-20

**Authors:** Melanie S. Brennan, Maria F. Matos, Bing Li, Xiaoping Hronowski, Benbo Gao, Peter Juhasz, Kenneth J. Rhodes, Robert H. Scannevin

**Affiliations:** 1 Department of Neurology Research, Biogen Idec, Inc., Cambridge, MA, United States of America; 2 Department of Discovery Proteomics and Mass Spectrometry, Biogen Idec, Inc., Cambridge, MA, United States of America; North Carolina State University, UNITED STATES

## Abstract

Delayed-release dimethyl fumarate (also known as gastro-resistant dimethyl fumarate), an oral therapeutic containing dimethyl fumarate (DMF) as the active ingredient, is currently approved for the treatment of relapsing multiple sclerosis. DMF is also a component in a distinct mixture product with 3 different salts of monoethyl fumarate (MEF), which is marketed for the treatment of psoriasis. Previous studies have provided insight into the pharmacologic properties of DMF, including modulation of kelch-like ECH-associated protein 1 (KEAP1), activation of the nuclear factor (erythroid-derived 2)-like 2 (NRF2) pathway, and glutathione (GSH) modulation; however, those of MEF remain largely unexplored. Therefore, the aim of this study was to evaluate the *in vitro* effects of DMF and MEF on KEAP1 modification, activation of the NRF2 pathway, and GSH conjugation. Using mass spectrometry, DMF treatment resulted in a robust modification of specific cysteine residues on KEAP1. In comparison, the overall degree of KEAP1 modification following MEF treatment was significantly less or undetectable. Consistent with KEAP1 cysteine modification, DMF treatment resulted in nuclear translocation of NRF2 and a robust transcriptional response in treated cells, as did MEF; however, the responses to MEF were of a lower magnitude or distinct compared to DMF. DMF was also shown to produce an acute concentration-dependent depletion of GSH; however, GSH levels eventually recovered and rose above baseline by 24 hours. In contrast, MEF did not cause acute reductions in GSH, but did produce an increase by 24 hours. Overall, these studies demonstrate that DMF and MEF are both pharmacologically active, but have differing degrees of activity as well as unique actions. These differences would be expected to result in divergent effects on downstream biology.

## Introduction

Multiple sclerosis (MS) is a chronic, progressive, autoimmune disease characterized by inflammation and neurodegeneration, and is the most common demyelinating disorder of the central nervous system (CNS) in young adults [1–3]. MS presents in several clinical forms including relapsing-remitting MS (RRMS), which is characterized by relapses (or flares) that eventually lead to increased disability, and is the predominant form, affecting approximately 80–85% of MS patients [1–3]. Although the exact mechanisms underlying the pathophysiology of RRMS remain to be determined, decreasing inflammation and promoting resistance to excessive oxidative stress may reduce the rate of disease progression, as both play a critical role in the neurodegenerative component of this debilitating disease [4–6].

**Fig 1:**
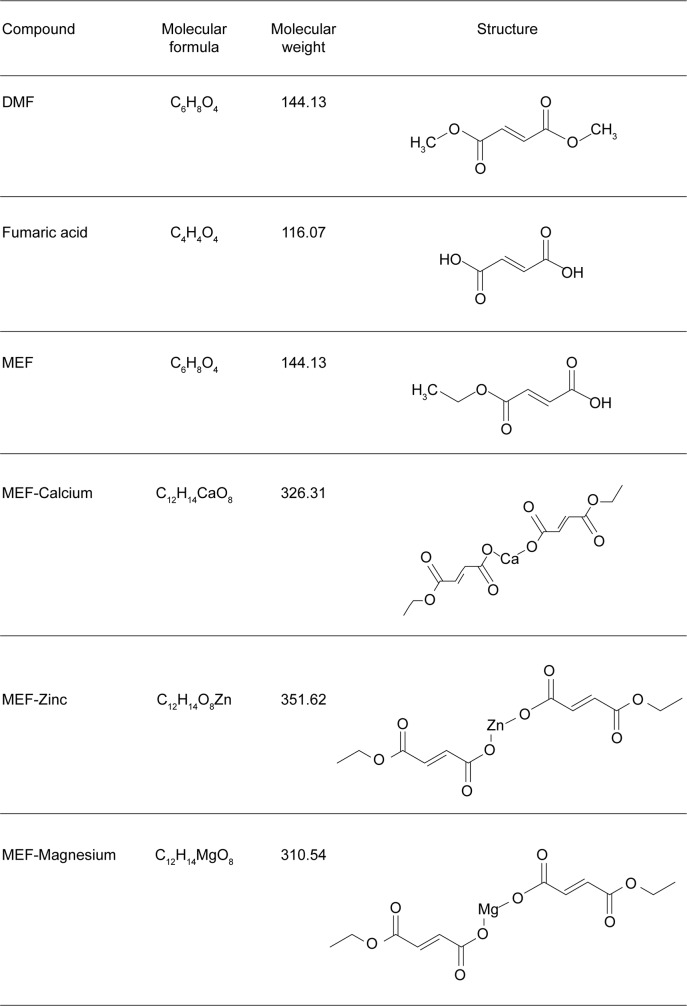
Structure and basic properties of DMF, MEF salts and Fumaric Acid. DMF and MEF are esters of fumaric acid, which is not pharmacologically active. The active moiety(ies) of DMF and MEF confer their chemical and physical properties.

Dimethyl fumarate (DMF; Fig. 1) has been shown to have anti-inflammatory, cytoprotective, and immunomodulatory properties in pre-clinical models of MS [7, 8]. A clinical formation of DMF (referred to as gastro-resistant DMF or delayed-release DMF) is approved in the United States, New Zealand and Australia for the treatment of relapsing forms and relapsing MS, respectively, and in the European Union, Switzerland and Canada for the treatment of RRMS based on clinical and radiological efficacy in patient clinical trials. The exact mechanisms by which gastro-resistant DMF exerts its clinical efficacy are unknown, but some of these effects are believed to be mediated through activation of the nuclear factor (erythroid-derived 2)-like 2 (NRF2) pathway, an endogenous defense mechanism against toxic cell stress [7, 8]. Under basal conditions, NRF2 is sequestered in the cytoplasm by the actin-associated protein kelch-like ECH-associated protein 1 (KEAP1), which targets NRF2 for ubiquitination and subsequent proteasomal degradation [9, 10]. However, in the presence of electrophiles or oxidative stress, these molecules can bind KEAP1 cysteine residues resulting in an allosteric conformational change that diminishes KEAP1-dependent degradation of NRF2 [11]. This allows NRF2 to accumulate and translocate to the nucleus to regulate cytoprotective genes associated with the phase II antioxidant response [12,13]. Various synthetic and naturally occurring compounds possessing electrophilic properties, including DMF, have been shown to modify specific cysteine residues on KEAP1 and subsequently activate NRF2 [14–18].

Along with research supporting NRF2 activation as a cytoprotective mechanism of DMF, other studies have hypothesized indirect regulation of NRF2 or NRF2-independent mechanisms of action for DMF, including activation of the hydroxycarboxylic acid receptor 2 (HCA2), inhibition of nuclear factor kappa-light-chain-enhancer of activated B cells (NF-κB), activation of hypoxia-inducible factor 1-alpha (HIF1A) and modulation of cellular glutathione (GSH) [19–22]. As α, β carboxylic acid unsaturated esters, fumarates are capable of interacting with various free cysteine residues by Michael addition including those present on the antioxidant thiol, GSH. Schmidt and colleagues have shown that DMF can stably bind GSH and rapidly deplete circulating levels [23]. Other groups have suggested that this intracellular depletion contributes to the anti-inflammatory, immunosuppressive and cytoprotective properties of DMF [24–28]. It is therefore likely that multiple mechanisms underlie the immunomodulatory and neuroprotective effects of DMF.

DMF together with three salt conjugates (Ca^2+^, Mg^2+^, Zn^2+^) of monoethyl fumarate (MEF) comprise a therapeutic for the treatment of moderate to severe forms of psoriasis [29]. Despite their demonstrated clinical utility the molecular mechanisms that underlie the therapeutic effects of fumaric acid ester compounds are largely unknown. It is also unknown if these chemically distinct fumarate esters, such as DMF and MEF, elicit different biological responses that would be expected to result in different pharmacokinetic and/or pharmacodynamic effects. Characterizing the pharmacodynamic properties of DMF and MEF will provide important insights into the mechanisms of action for different fumaric acid ester compounds. The aim of this study was to evaluate the *in vitro* properties of DMF and MEF in the context of KEAP1 modification, NRF2 activation, and GSH depletion to determine if the two share common or distinct characteristics.

## Materials and Methods

### Compound Handling

DMF and MEF were prepared as 30 mg/mL solutions in dimethyl sulfoxide (DMSO), titrated in DMSO, and then diluted into normal growth media for cell treatments. MEF salt conjugates contain 2 MEF molecules joined by a single divalent cation (Ca^2+^, Mg^2+^ or Zn^2+^) that hydrolyzes to 2 free MEFs plus the inorganic salt at neutral pH. Utilizing a weight per unit volume measure (μg/mL) allows for approximately equal amounts of total fumaric acid ester to be added for both the MEF salt conjugates and DMF in these assay conditions. The MEF ester mixture was made by combining 87 mg MEF-Ca^2+^, 5 mg MEF-Mg^2+^ and 3 mg MEF-Zn^2+^ in the appropriate volume of DMSO to yield a total concentration of 30 mg/mL. The final concentration of DMSO (0.04%) was consistent for all treated cells.

### Analysis of KEAP1 Modification

Low passage number human embryonic kidney 293 (HEK293FT) (Life technologies, Grand Island, NY) cells were transfected with pFRT-Keap1-V5 (rat KEAP1 with C-terminal V5 and His6 tags). Two days post-transfection, cells were treated with 3 and 6 μg/mL DMF or MEF salts (Ca^2+^, Mg^2+^, Zn^2+^) diluted in DMSO or DMSO as a control for 6 hours. Tagged KEAP1 was then immunoprecipitated with anti-V5 agarose (Sigma, St. Louis, MO), fractionated by sodium dodecyl sulfate-polyacrylamide gel electrophoresis (SDS-PAGE), detected with Coomassie Brilliant Blue (Bio-Rad, Hercules, CA), and bands corresponding to KEAP1 excised from the gel. The gel slice was reduced by dithiothreitol (DTT), alkylated by iodoacetamide and digested with trypsin. Resultant peptide pools were separated on a Dionex C18 column and analyzed on a Thermo Fisher Scientific LTQ FT Ultra Hybrid mass spectrometer and a Q-Exactive mass spectrometer (Waltham, MA). Mascot Server version 2.4 (Matrix Science, Boston, MA) was used to identify KEAP1 peptides and cysteine modifications. Carbamidomethylation or dimethyl- or monoethyl- or monomethyl-succination (due to partial de-methylation of DMF) were considered as variable cysteine modifications with allowance for methanol or ethanol or ammonia loss from N-terminal cysteines. Ion intensities of various succinated peptides were normalized to the summed ion intensities of all forms of cysteine-containing peptides to generate residue-specific values for conversion rates by fumarates.

### NRF2 Translocation Assay and Western Blot

Primary human spinal cord astrocytes (ScienCell, San Diego, CA) were treated for 6 hours with 1, 3, or 6 μg/mL of DMF or a mixture of MEF salts (Ca^2+^, Mg^2+^, Zn^2+^) or DMSO as a vehicle control. Cytosolic and nuclear fractions were prepared by using a nuclear extract kit from Active Motif (Carlsbad, CA). Total protein from cell fractions was quantified using the Pierce BCA protein assay (Thermo Fisher Scientific) and samples were diluted to equal concentrations. Nuclear translocation of NRF2 was analyzed using the TransAM NRF2 ELISA assay according to the manufacturer’s protocol (Active Motif). Cell extracts were also analyzed by immunoblotting with antibodies against NRF2 (Epitomics, Burlingame, CA), β-actin (MP Biomedicals, Santa Ana, CA) and histone deacetylase (HDAC1) (Cell Signaling, Danvers, MA). HDAC1 and β-actin were used as loading controls to verify equal protein in each lane and HDAC1 was also used to determine purity of the nuclear fraction. Immunoblots were quantitated by densitometry and graphed data represents one of three experimental repeats.

### *In Vitro* Gene Expression

Primary cultures of human spinal cord astrocytes were treated in triplicate with DMF, a mixture of MEF salts (Ca^2+^, Mg^2+^, Zn^2+^), fumaric acid (0, 1, 3, 6, 12 μg/mL) or DMSO control for 24 hours followed by RNA extraction using RNeasy 96 plates. Samples were purified by the spin method according the manufacturer’s protocol (RNeasy 96 Universal Protocol, QIAgen, Hilden Germany). Samples were reverse-transcribed into cDNA according to the manufacturer protocols (Life Technologies, Carlsbad, CA) and analyzed by real-time polymerase chain reaction (RT-PCR). Human target gene primers for glyceraldehyde-3-phosphate dehydrogenase (*GAPDH*): Hs02758991_g1; oxidative stress induced growth inhibitor 1 (*OSGIN1*): Hs00203539_m1; glutamate cysteine ligase, catalytic subunit (*GCLC*): Hs00155249_m1; heme oxygenase 1 (*HMOX1*): Hs01110250_m1; NAD(P)H dehydrogenase (quinone 1) (*NQO1*): Hs02512143_s1; sulfiredoxin-1 (*SRXN1*): Hs00607800_m1; thioredoxin reductase 1, cytoplasmic (*TXNRD1*): Hs00182418_m1 and 6-FAM dye-labeled TaqMan MGB probes (Life Technologies). Reactions containing 100 ng of cDNA, 900 nM of each primer, and 250 nM TaqMan probes were cycled on a QuantStudio 12k-flex system (Life Technologies) once for 10 min at 95°C, followed by 40 cycles of 95°C for 10 s and 60°C for 1 min. All samples were measured in triplicate with *GAPDH* as a normalizing gene. Comparative C_T_ method was used to calculate fold changes and samples were normalized relative to vehicle control conditions. Graphed data represents one of three experimental repeats.

### GSH Analysis

Primary cultures of human spinal cord astrocytes were treated in triplicate with DMF, salt forms of MEF (Ca^2+^, Mg^2+^, Zn^2+^), fumaric acid (0, 1, 3 μg/mL), or DMSO control. Replicate cultures were treated such that cells were exposed to compound for 0.5, 1, 6, 12, or 24 hours. Cellular and extracellular GSH levels were measured using the GSH-Glo^TM^ Glutathione Luminescence Assay according to the manufacturer’s protocol (Promega, Madison WI). Media from treated cells was used for extracellular GSH analysis using the tissue extracts protocol. Total relative luminescence units (RLU) are graphed as means ± SD. Graphed data represents one of three experimental repeats.

## Results

### KEAP1 cysteine residues are differentially modified by DMF versus MEF

**Fig 2:**
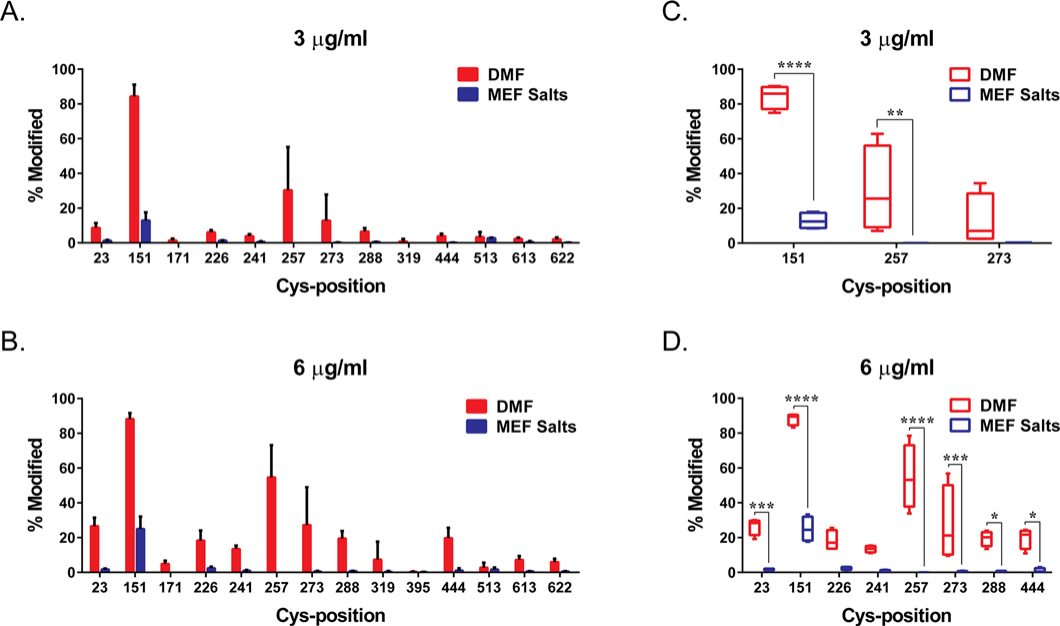
DMF and MEF Differentially Modify KEAP1 Cysteine Residues. KEAP1 transfected HEK 293FT cells were treated with DMF and MEF salts (Ca^2+^, Mg^2+^, Zn^2+^) at 3 μg/mL (A, C) or 6 μg/mL (B, D). Resulting cysteine modifications on KEAP1 were analyzed using mass spectrometry. Percent modification of KEAP1 cysteine residues with DMF or MEF was determined relative to DMSO control treated cells. (A, B) Representation of percent cysteine modification of analyzed KEAP1 cysteine residues in the presence of 3 (A) or 6 (B) μg/mL DMF or MEF. Each bar represents the means of quadruplicate determinations (± SD). (C, D) Box-whisker plots demonstrate the means, quartiles, and max-min of KEAP1 cysteine residues modified by greater than 10 percent in A and B. *, *p*<0.05. ***, *p*<0.001. ****, *p*<0.0001. *P* values are based on two-way analysis of variance (ANOVA) with Sidak’s post-test for multiple comparisons.

Under basal conditions, NRF2 is bound to the repressor protein, KEAP1, which targets NRF2 for degradation by the ubiquitin proteasome pathway. Specific modifications of KEAP1 cysteine residues by electrophiles and various compounds, including DMF, have been shown to result in inhibition of NRF2 degradation, allowing NRF2 to translocate to the nucleus and regulate gene expression [30]. Cysteine 151 (Cys151) modification of KEAP1 has been identified as one of the major sites important for stress sensing; however, modifications of other KEAP1 cysteine residues, including but not limited to cysteine 257 (Cys257), cysteine 273 (Cys273) and cysteine 288 (Cys288), have also been identified to contribute to KEAP1-dependent stabilization of NRF2 [31,32]. Using mass spectrometry, modification of KEAP1 cysteine residues were analyzed in the presence of different concentrations of DMF or MEF salts. Both 3 μg and 6 μg DMF were able to modify KEAP1 cysteine residues by greater than 10 percent, respectively, for Cys151 (84.3%; 88.2%), Cys257 (30.3%; 54.7%) and Cys273 (12.8%; 27.2%; Fig. 2). Modification of Cys151 (12.9%; 23.5%) was also observed following treatment with both 3 μg and 6 μg, respectively, of MEF; however, the overall degree of modification was significantly less compared with DMF-treated cells (Figs. 2C and 2D). No significant modification of Cys257 and Cys273 was identified after treatment with MEF.

### DMF induces a greater magnitude of nuclear NRF2 translocation compared to MEF

**Fig 3:**
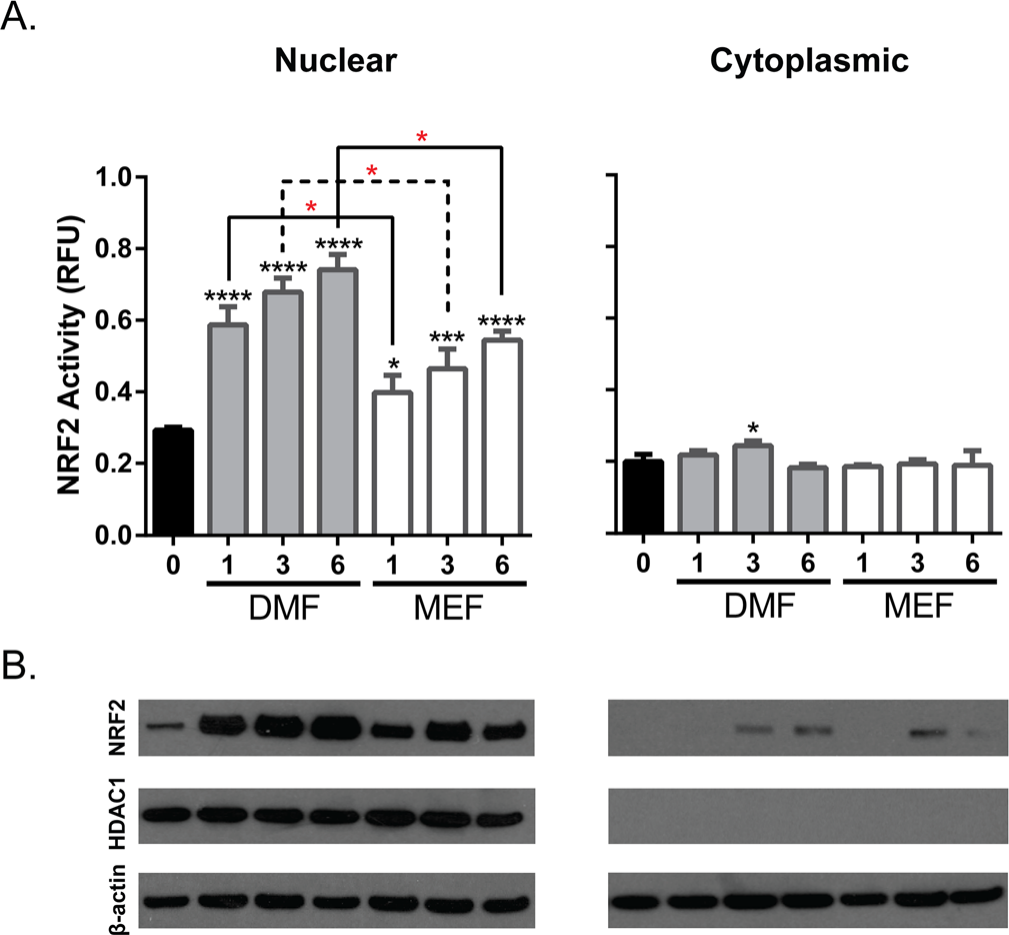
NRF2 Translocation Induced by DMF and MEF Treatment. Human astrocytes were treated with a mixture of MEF salts (Ca^2+^, Mg^2+^, Zn^2+^), DMF or the vehicle control DMSO. Extracts from harvested cells were separated into nuclear and cytoplasmic fractions and equal protein amounts from triplicate aliquots of each fraction were analyzed using an NRF2 TransAM DNA binding assay (A), and Western blots for NRF2, HDAC1 or β-actin (B). Red asterisks (*, *p*<0.01) indicate comparison of DMF versus MEF at indicated concentration. Black asterisks directly above bar plots (*, *p*<0.05, ***, *p*<0.001, ****, *p*<0.0001) refer to DMF and MEF versus DMSO control at indicated concentrations. *P* values are based on two-way ANOVA with Tukey’s post-test for multiple comparisons.

Following the modification of KEAP1 cysteine residues, the conformation of KEAP1 is altered, resulting in decreased NRF2 degradation, subsequent nuclear translocation and induction of target gene expression [33]. Based on the differential modification of KEAP1 observed with DMF and MEF, investigation into the effect of these compounds on NRF2 protein accumulation was conducted. To determine whether DMF or a combination of the three salt conjugates of MEF were capable of inducing accumulation of the NRF2 protein, human astrocytes were treated for 6 hours with DMF, MEF salts or DMSO control and total levels of NRF2 protein were analyzed in nuclear and cytoplasmic cell fractions. Both DMF and the salt conjugates of MEF were able to induce nuclear and cytoplasmic accumulation of NRF2 protein compared with DMSO controls in a concentration dependent manner as assessed by both NRF2 DNA binding assays (Fig. 3A) and Western blotting (Fig. 3B). NRF2 protein accumulation occurred mostly within the nuclear fraction of these cells, and accumulation of NRF2 in the nuclei of DMF treated cells compared to vehicle control was 2.0, 2.3 and 2.5 fold greater following 1, 3 and 6 μg compound, respectively. Nuclear accumulation of NRF2 in DMF treated cells was significantly greater than MEF treated cells; 1.4, 1.6 and 1.9 fold greater than vehicle control for 1, 3 and 6 μg compound, respectively (Fig. 3A). Purity of the nuclear fraction was confirmed using the nuclear-specific protein HDAC1 and β-actin was included as a cytoplasmic loading control (Fig. 3B).

### DMF and MEF produce distinct changes in gene expression

**Fig 4:**
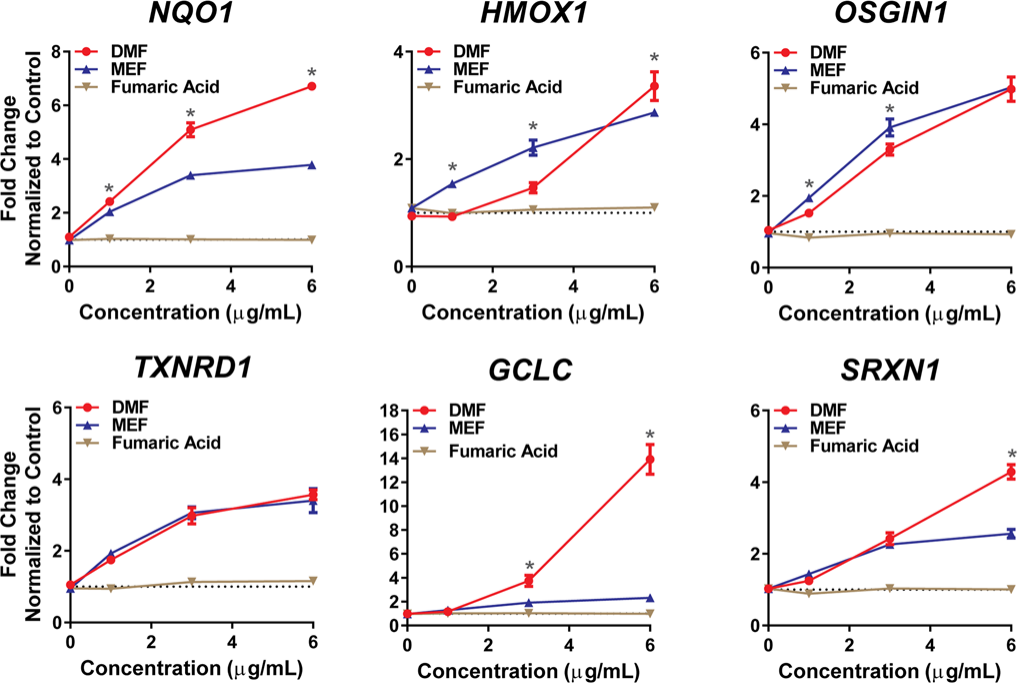
DMF and MEF Produce Distinct Changes in Gene Expression in Astrocytes. Human astrocytes were treated with a concentration-response of DMF, MEF salts (Ca^2+^, Mg^2+^, Zn^2+^) or fumaric acid for 24 hours, and analyzed for transcriptional changes in putative NRF2 target genes (*NQO1*, *HMOX1*, *OSGIN1*, *TXNRD1*, *GCLC* and *SRXN1*). Triplicate determinations (± SD) were normalized as a fold change relative to DMSO controls for each gene and probe set. *, *p*<0.01 for DMF versus MEF at indicated concentration. *P* values are based on two-way ANOVA with Tukey’s post-test for multiple comparisons.

Since the nuclear accumulation of NRF2 should result in NRF2-dependent transcriptional gene regulation [13,34], expression of several NRF2-target genes was assessed after treatment with DMF and the combination of MEF salts in human astrocytes. Gene expression in cells exposed to fumaric acid alone was also analyzed within these studies as a control. Both DMF and MEF induced distinct patterns of concentration-dependent changes in gene expression for all genes analyzed (Fig. 4). At higher concentrations of DMF or MEF treatment (6 μg), *NQO1*, *HMOX1*, *GCLC* and *SRXN1* were induced to a greater extent with DMF compared with MEF (Fig. 4). In comparison, at lower concentrations (1 and 3 μg), MEF induced *HMOX1* and *OSGIN1* gene expression to a greater extent relative to DMF. Fumaric acid had no effect on gene expression. These results indicate that different esters of fumaric acid induce differential effects directly on gene transcription and potentially downstream regulatory processes that impact mRNA stability and accumulation. Statistical analysis of DMF and MEF versus DMSO control is provided in S1 Fig.

### DMF and MEF differentially regulate cellular GSH

**Fig 5:**
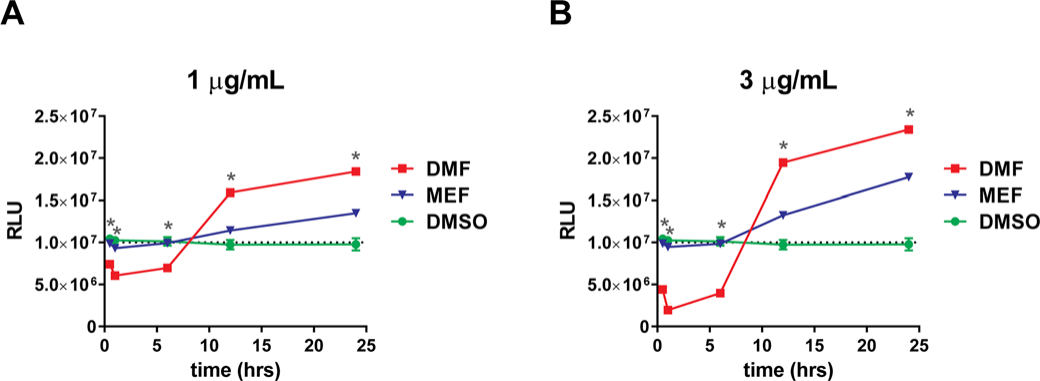
DMF and MEF Induce Different Effects on Cellular GSH. Primary cultures of human astrocytes were incubated with 1 (A) or 3 (B) μg/mL DMF, MEF, or DMSO as a control. Treated cells were harvested after 0.0, 0.5, 1.0, 6, 12, and 24 hours of treatment, and total cellular GSH was measured as relative luminescence units (RLU). Each point represents the mean of triplicate determinations (± SD). Dotted line represents average basal RLU levels. *, *p*<0.01 for DMF versus MEF at indicated time point. *P* values are based on two-way ANOVA with Tukey’s post-test for multiple comparisons.

**Fig 6:**
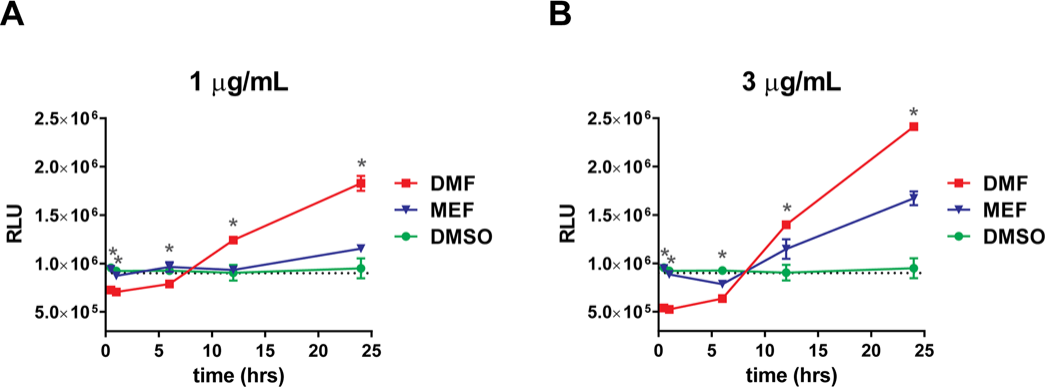
DMF and MEF Induce Different Effects on Extracellular GSH. Primary cultures of human astrocytes were incubated with 1 (A) or 3 (B) μg/mL DMF, MEF or DMSO as a control. Media was collected from treated cells after 0.0, 0.5, 1.0, 6, 12 or 24 hours of treatment, and total extracellular glutathione was measured as relative luminescence units (RLU). Each point represents the mean of triplicate determinations (± SD). Dotted line represents average basal RLU levels. *, *p*<0.01 for DMF versus MEF at indicated time point. *P* values are based on two-way ANOVA with Tukey’s post-test for multiple comparisons.

Although the data presented above support DMF activation of the NRF2 pathway in a KEAP1-dependent manner, other pathways or alternative mechanisms of activation of NRF2 may play a key role in the cellular responses to DMF, including GSH depletion [20]. GSH is the most abundant cellular thiol and kinetic studies of the GSH/DMF interaction have shown that DMF can stably conjugate with GSH and rapidly deplete free circulating GSH levels [23]. To determine whether treatment with DMF and MEF result in differential depletion of GSH, we analyzed total levels of intracellular and extracellular GSH in human astrocytes. DMF induced a concentration-dependent transient depletion of intracellular GSH that lasted approximately 10 hours and recovered by 12 hours. Following recovery from depletion, GSH continued to increase above basal levels (Fig. 5 and S2 Fig.). By 24 hours, total GSH levels following DMF treatment had increased approximately 2.0 fold and 2.5 fold above baseline for 1 or 3 μg/mL treatments, respectively (Figs. 5A and 5B). Measurement of extracellular GSH following DMF treatment resulted in similar depletion and recovery as observed for intracellular levels, with depletion occurring at early time points and increasing above baseline by 24 hours (Fig. 6 and S3 Fig.). In contrast, MEF did not deplete intracellular or extracellular GSH at any time point analyzed at either 1 or 3 μg/mL (Figs. 5A and 5B). However, MEF addition did result in a significant, but smaller increase in total intracellular and extracellular GSH levels above baseline at 24 hours, relative to DMF (Figs. 5 and 6). Fumaric acid had no effect on GSH levels (S4 Fig.). These results indicate that different esters of fumaric acid induce differential effects on GSH depletion and accumulation.

## Discussion

Fumaric acid esters have been used to treat psoriasis for over 20 years as a therapy containing a combination of DMF and three salt forms of MEF. More recently, a therapeutic containing only DMF, independent of MEF, was approved for the treatment of relapsing forms of MS. The use of these fumaric acid esters as promising therapies for autoimmune and neurodegenerative diseases has prompted interest in whether different fumaric acid esters exhibit similar or distinct mechanisms associated with their therapeutic actions. Despite the physical and chemical differences between DMF and MEF, unique biological responses induced by treatment with these fumaric acid esters have not yet been explored. Since activation of the NRF2 pathway and GSH modulation have been previously identified to contribute to the cytoprotective properties of DMF, regulation of these biological mechanisms was investigated for DMF and compared to responses for MEF. These studies demonstrate that both DMF and MEF are active *in vitro*; however, these data also support the conclusion that DMF and MEF display distinct and differential activities associated with activation of the NRF2 transcriptional pathway and GSH modulation.

Under basal conditions, KEAP1 inhibits NRF2-dependent transcription, effectively keeping endogenous expression levels of genes associated with the phase II antioxidant response at low levels. However, modification of the cysteine redox sensors on KEAP1, as a result of oxidation or by conjugation with electrophiles, results in NRF2 accumulation and transcriptional pathway activation [35]. Cys151, Cys273 and Cys288 have been recognized in the literature as major KEAP1 sensors and studies have suggested that different cysteine residues may play discrete functions mediating the fate of KEAP1 and phase II antioxidant regulation [36]. For example, Cys151 is critical for inhibition of KEAP1-dependent degradation of NRF2 by certain chemical inducers while Cys273 and Cys288 are required for NRF2 degradation via ubiquitination [10, 37–39]. Our findings show that DMF and MEF differentially modulate specific KEAP1 cysteine residues, with DMF treatment resulting in a more robust cysteine modification and targeting cysteines that span across the KEAP1 protein. The observation that MEF solely modulates Cys151 and to a significantly lesser degree compared to DMF, suggests that DMF may either differentially induce NRF2 activation or activate this pathway to a greater extent than MEF. Indeed, we have shown in human astrocytes that DMF induces significantly greater NRF2 protein accumulation compared to MEF, and NRF2 target genes are differentially regulated between these fumaric acid esters. However, despite the more robust induction of KEAP1 modification and NRF2 nuclear accumulation with DMF, the comparison of DMF versus MEF gene transcription is not straightforward. For example, DMF-mediated regulation of *OSGIN1* and *HMOX1* transcripts is significantly less robust at low concentrations compared to MEF even though DMF-induced NRF2 activation is greater at these same concentrations. This observation suggests that there are alternate pathways contributing to gene regulation following addition of DMF and MEF. Overall, these findings are consistent with the hypothesis that these compounds are both pharmacologically active, but have differing degrees of NRF2 activation as well as alternate pathway regulation, which may result in divergent modulation of downstream biological functions. Further investigation into the importance of differential KEAP1 cysteine modification and NRF2 gene regulation in the presence of DMF and MEF is necessary to understand the impact of divergent NRF2 regulation on cellular biology.

In addition to activation of the NRF2 pathway, fumaric acid esters have been hypothesized to activate NRF2-independent mechanisms, and an example of the latter may be in the modulation of cellular GSH [20]. GSH is known to play an important role in cellular defense against various stressors, and the regulation of GSH in astrocytes has shown to directly benefit neuronal health [40]. Interestingly, more recent data suggests that GSH depletion can be protective against inflammation and neurodegeneration [41–43]. Furthermore, studies have suggested that GSH depletion is responsible for the anti-inflammatory and immunomodulatory effects of DMF by inducing the stress response protein *HMOX1*, leading to reductions in inflammatory cytokine secretion and potentially a secondary antioxidative response [24]. Additionally, it has also been shown that DMF- dependent GSH depletion leads to the induction of type II dendritic cells *in vivo* through modulation of *HMOX1* and Signal Transducer and Activator of Transcription 1 (*STAT1*) silencing [25]. Our data obtained in astrocytes demonstrates that while treatment with DMF resulted in a robust effect on GSH depletion, both within the intracellular and extracellular space, MEF did not deplete GSH, suggesting that DMF may modulate additional protective pathways compared to MEF and potentially induce anti-inflammatory machinery to a greater extent. In contrast to GSH depletion, both compounds did result in an accumulation of intracellular and extracellular GSH at later time points, even though total GSH levels were higher following DMF treatment. These increases in total GSH following initial depletion may be a compensatory response to cellular GSH loss in the presence of DMF and this may play a protective role; however, a more direct role of NRF2-dependent upregulation of GSH biosynthetic machinery is also possible since both fumaric acid esters eventually increased GSH levels above baseline [26]. The ability of DMF to deplete GSH levels in comparison to MEF supports a distinct mechanism of action for DMF, potentially due to the more reactive structure of DMF compared to MEF; however, further exploration is necessary to understand the importance of this unique DMF-specific response.

Taken together, our studies indicate that fumaric acid esters may have significantly different biochemical properties that divergently impact cellular pathways, including activation of the NRF2 pathway and modulation of cellular GSH. Based on these findings, it would be expected that these *in vitro* differences would manifest in different pharmacodynamic and pharmacokinetic properties *in vivo*. The clinical consequences of these differences remain to be explored.

## Supporting Information

S1 Fig DMF and MEF Produce Distinct Changes in Gene Expression in Astrocytes: Statistical Analysis for DMF and MEF Compared to DMSO Control. Human astrocytes were treated with a concentration-response of DMF, MEF salts (Ca^2+^, Mg^2+^, Zn^2+^) or fumaric acid for 24 hours, and analyzed for transcriptional changes in putative NRF2 target genes (*NQO1*, *HMOX1*, *OSGIN1*, *TXNRD1*, *GCLC* and *SRXN1*). Triplicate determinations (± SD) were normalized as a fold change relative to DMSO controls for each gene and probe set. *, *p*<0.01 for DMF (red) and MEF (blue) versus DMSO at indicated concentrations. *P* values are based on one-way ANOVA with Dunnett’s post-test for multiple comparisons.
(TIF)

S2 Fig DMF and MEF Induce Different Effects on Cellular GSH: Statistical Analysis for DMF and MEF Compared to DMSO Control. Primary cultures of human astrocytes were incubated with 1 (A) or 3 (B) μg/mL DMF, MEF, or DMSO as a control. Treated cells were harvested after 0.0, 0.5, 1.0, 6, 12, and 24 hours of treatment, and total cellular GSH was measured as relative luminescence units (RLU). Each point represents the mean of triplicate determinations (± SD). Dotted line represents average basal RLU levels. *, *p*<0.01 for DMF (red) and MEF (blue) versus DMSO control at indicated time points. *P* values are based on two-way ANOVA with Tukey’s post-test for multiple comparisons.
(TIF)

S3 Fig DMF and MEF Induce Different Effects on Extracellular GSH: Statistical Analysis for DMF and MEF Compared to DMSO Control. Primary cultures of human astrocytes were incubated with 1 (A) or 3 (B) μg/mL DMF, MEF or DMSO as a control. Media was collected from treated cells after 0.0, 0.5, 1.0, 6, 12 or 24 hours of treatment, and total extracellular glutathione was measured as relative luminescence units (RLU). Each point represents the mean of triplicate determinations (± SD). Dotted line represents average basal RLU levels. *, *p*<0.01 for DMF (red) and MEF (blue) verus DMSO control at indicated time points. *P* values are based on two-way ANOVA with Tukey’s post-test for multiple comparisons.
(TIF)

S4 Fig DMF, MEF and Fumaric Acid Induce Different Effects on Cellular GSH. Primary cultures of human astrocytes were incubated with 1 (A) or 3 (B) μg/mL DMF, MEF, fumaric acid or DMSO as a control. Treated cells were harvested after 0.0, 0.5, 1.0, 6, 12, and 24 hours of treatment, and total cellular GSH was measured. Each point represents the mean of triplicate determinations (± SD). Dotted line represents average basal GSH levels. *, *p*<0.01 for DMF versus MEF at indicated time point. *P* values are based on two-way ANOVA with Tukey’s post-test for multiple comparisons.
(TIF)
