# Supplementary figures and images for: Dimethyl Fumarate and Monoethyl Fumarate Exhibit Differential Effects on KEAP1, NRF2 Activation, and Glutathione Depletion In Vitro
Source: PLoS One. 2015 Mar 20;10(3):e0120254. doi: 10.1371/journal.pone.0120254 (PMC4368598; doi:10.1371/journal.pone.0120254)

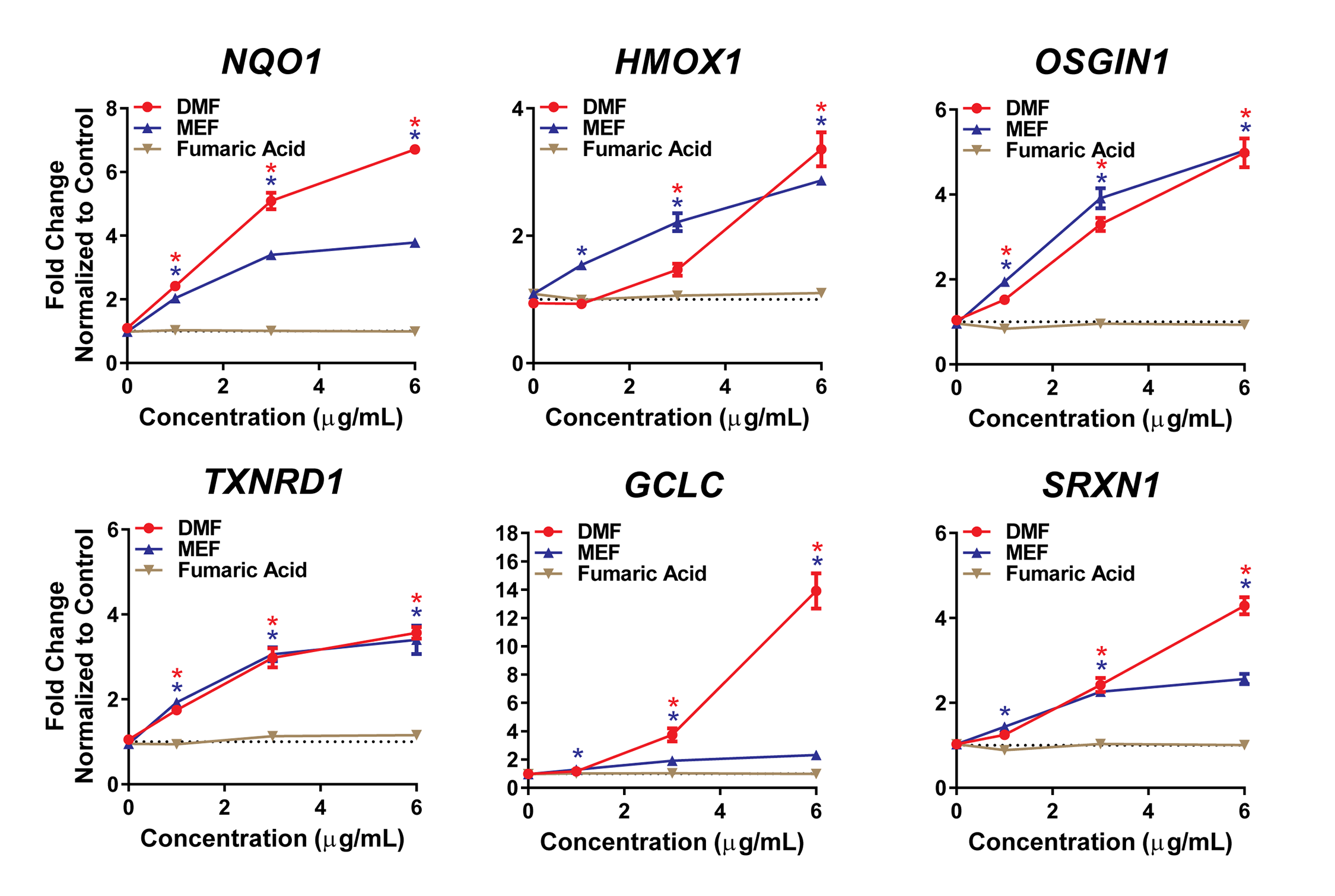

Supplement: S1 Fig — Human astrocytes were treated with a concentration-response of DMF, MEF salts (Ca2+, Mg2+, Zn2+) or fumaric acid for 24 hours, and analyzed for transcriptional changes in putative NRF2 target genes (NQO1, HMOX1, OSGIN1, TXNRD1, GCLC and SRXN1). Triplicate determinations (± SD) were normalized as a fold change relative to DMSO controls for each gene and probe set. *, p<0.01 for DMF (red) and MEF (blue) versus DMSO at indicated concentrations. P values are based on one-way ANOVA with Dunnett’s post-test for multiple comparisons. (TIF) [file pone.0120254.s001.tif]

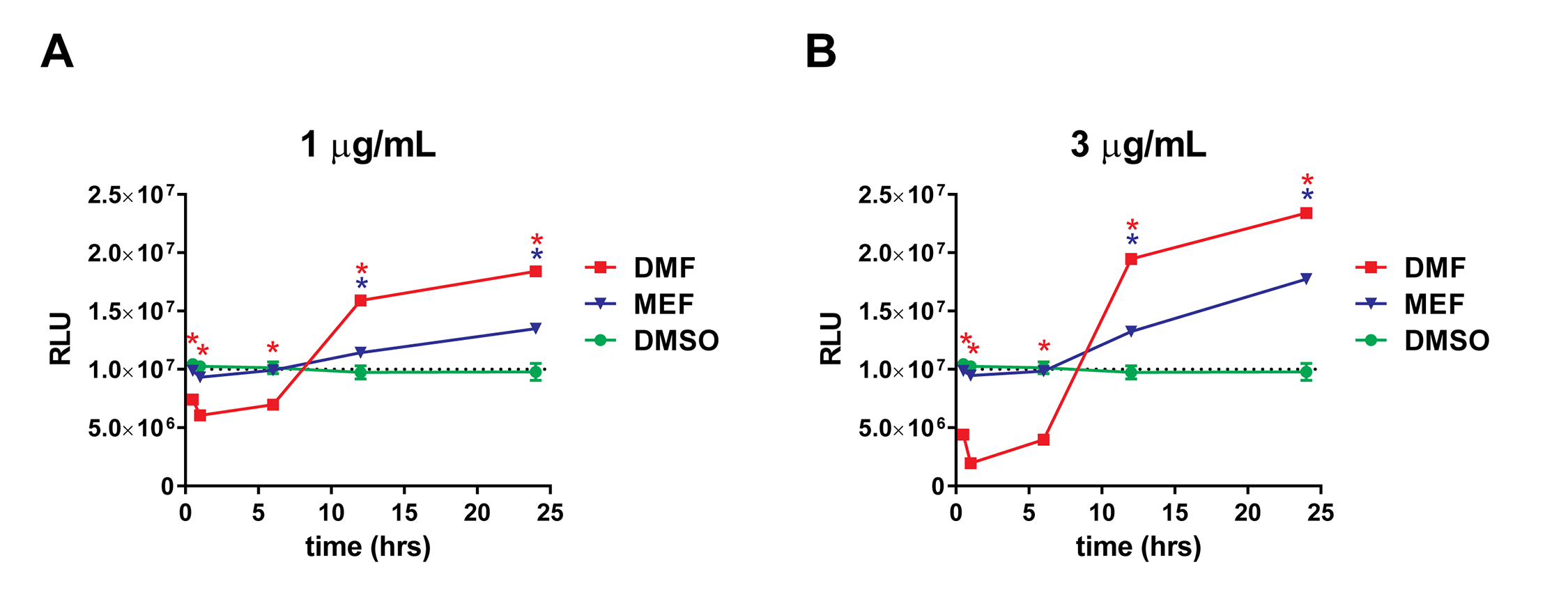

Supplement: S2 Fig — Primary cultures of human astrocytes were incubated with 1 (A) or 3 (B) μg/mL DMF, MEF, or DMSO as a control. Treated cells were harvested after 0.0, 0.5, 1.0, 6, 12, and 24 hours of treatment, and total cellular GSH was measured as relative luminescence units (RLU). Each point represents the mean of triplicate determinations (± SD). Dotted line represents average basal RLU levels. *, p<0.01 for DMF (red) and MEF (blue) versus DMSO control at indicated time points. P values are based on two-way ANOVA with Tukey’s post-test for multiple comparisons. (TIF) [file pone.0120254.s002.tif]

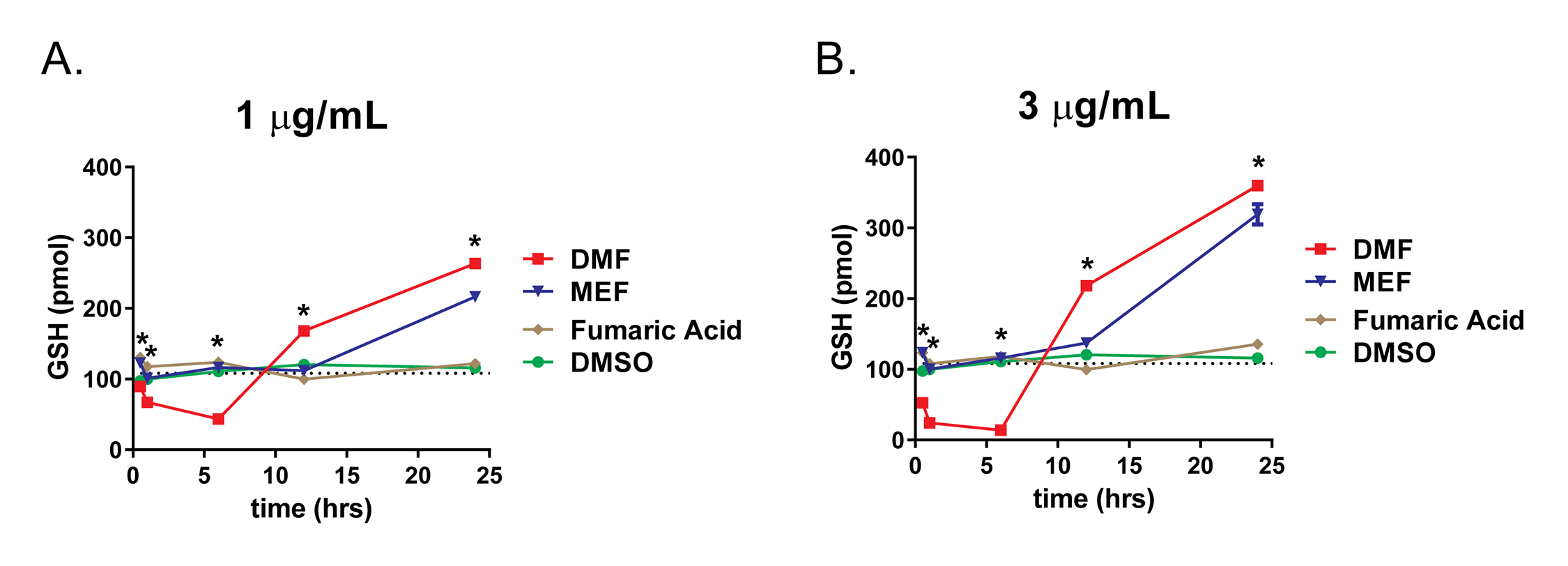

Supplement: S3 Fig — Primary cultures of human astrocytes were incubated with 1 (A) or 3 (B) μg/mL DMF, MEF or DMSO as a control. Media was collected from treated cells after 0.0, 0.5, 1.0, 6, 12 or 24 hours of treatment, and total extracellular glutathione was measured as relative luminescence units (RLU). Each point represents the mean of triplicate determinations (± SD). Dotted line represents average basal RLU levels. *, p<0.01 for DMF (red) and MEF (blue) verus DMSO control at indicated time points. P values are based on two-way ANOVA with Tukey’s post-test for multiple comparisons. (TIF) [file pone.0120254.s003.tif]

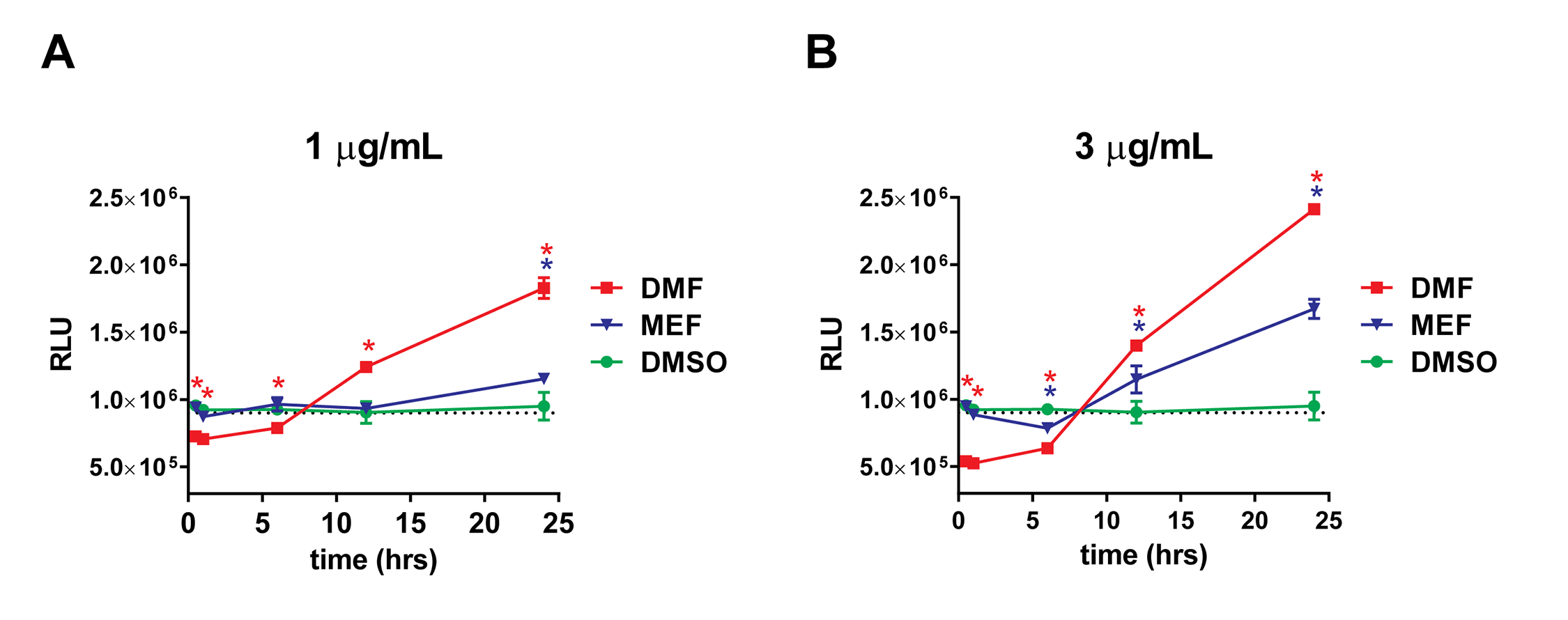

Supplement: S4 Fig — Primary cultures of human astrocytes were incubated with 1 (A) or 3 (B) μg/mL DMF, MEF, fumaric acid or DMSO as a control. Treated cells were harvested after 0.0, 0.5, 1.0, 6, 12, and 24 hours of treatment, and total cellular GSH was measured. Each point represents the mean of triplicate determinations (± SD). Dotted line represents average basal GSH levels. *, p<0.01 for DMF versus MEF at indicated time point. P values are based on two-way ANOVA with Tukey’s post-test for multiple comparisons. (TIF) [file pone.0120254.s004.tif]
